# Impact of endogenous viral elements on glioma clinical phenotypes by inducing OCT4 in the host

**DOI:** 10.3389/fcimb.2024.1474492

**Published:** 2024-11-11

**Authors:** Shirong Li, Shuai He, Haoyu Xue, Yi He

**Affiliations:** ^1^ Department of Neurosurgery and Laboratory of Animal Tumor Models, Cancer Center and Frontiers Science Center for Disease-related Molecular Network, West China Hospital, Sichuan University, Chengdu, Sichuan, China; ^2^ The First Dongguan Affiliated Hospital, Guangdong Medical University, Dongguan, China; ^3^ Department of Neurosurgery, Shenzhen Key Laboratory of Neurosurgery, The First Affiliated Hospital of Shenzhen University, Shenzhen Second People’s Hospital, Shenzhen, China

**Keywords:** endogenous viral elements, RNA, transcription factors, OCT4, glioblastoma, glioblastoma stem cells

## Abstract

**Introduction:**

Endogenous viral elements (EVEs) are viral sequences integrated within the host genome that can influence gene regulation and tumor development. While EVEs have been implicated in cancer, their role in regulating key transcription factors in glioblastoma (GBM) remains underexplored. This study investigates the relationship between EVEs and the activation of OCT4, a critical transcription factor in GBM progression.

**Methods:**

We utilized CancerHERVdb and HervD Atlas databases to identify potential interactions between EVEs and key genes involved in GBM. Data from 273 GBM patient samples in the TCGA database were analyzed to examine the correlation between OCT4 expression and mutations in glioma-related genes. Furthermore, glioblastoma stem cells (GSCs) were assessed for the expression levels of OCT4 and SOX2, and Pearson correlation analysis was performed.

**Results:**

Our analysis revealed that OCT4 is a pivotal gene activated by EVEs in GBM. OCT4 expression was significantly correlated with mutations in key glioma-associated genes. Higher OCT4 levels were associated with poorer patient prognosis, higher tumor grades, and older age. Additionally, GSCs exhibited elevated expression of both OCT4 and SOX2, with a positive correlation observed between these two genes in GBM patients.

**Discussion:**

This study highlights the potential role of EVEs in driving GBM progression through the activation of OCT4. The findings emphasize the importance of OCT4 in GBM malignancy and suggest that targeting EVE-mediated pathways may provide new therapeutic approaches for GBM treatment.

## Introduction

In nature, there are extensive interaction mechanisms between microorganisms and hosts (Thompson et al., 2020; Bao et al., 2024; Chu et al., 2024). Endogenous viruses, in particular, integrate into the human genome during these interactions and occupy a significant portion of it (Paces et al., 2004; Katzourakis and Gifford, 2010; Li et al., 2024). Endogenous viral elements (EVEs) refer to ancient viral genetic sequences that have been integrated and preserved in the host genome throughout evolutionary history. These elements date back millions of years, originating from viruses that once actively integrated into the host DNA (Holmes, 2011). In the human genome, these EVEs account for more than 8% (Lander et al., 2001; Li et al., 2001; Nurk et al., 2022). The presence of EVEs in the host genome not only unveils the intricate co-evolutionary history between hosts and viruses but also plays significant roles in biological processes, including gene regulation, expression patterns, and immune responses (Feschotte and Gilbert, 2012). For instance, some EVEs can influence host cellular functions by producing non-coding RNAs or regulating the expression of other genes, thereby playing critical roles in genomic stability and adaptive evolution (Katzourakis and Gifford, 2010; Ramsoomair et al., 2023). Moreover, research has indicated that endogenous viruses can impact cancer progression (Jansz and Faulkner, 2021). Human Endogenous Retroviruses (HERVs) constitute the majority of EVEs. The selective upregulation of HERV-E in clear cell renal carcinoma (Cherkasova et al., 2016) and the upregulation of HERV-H in colorectal cancer (Liang et al., 2012) may be related to tumor progression. In acute myeloid leukemia (AML), HERV-K is a source of enhancers with oncogenic potential (Deniz et al., 2020). HERV-K promotes tumor development by inducing breast cancer migration and invasion through the activation of the ERK pathway (Lemaître et al., 2017). HERV-derived long terminal repeat (LTR) sequences generate long non-coding RNAs that can promote the progression of triple-negative breast cancer (TNBC) and serve as potential therapeutic targets (Jin et al., 2019). Human endogenous retrovirus proteins Np9 and Rec may function oncogenically by derepressing c-myc through the inhibition of PLZF (Denne et al., 2007).

Glioblastomas are among the most common types of malignant brain tumors, known for their high invasiveness, treatment resistance, and poor prognosis (Jiang et al., 2024; Lin et al., 2024). These tumors originate from glial cells, which support nerve cells, and can be classified into various subtypes such as astrocytomas and oligodendrogliomas based on their origin, mutation profiles, and biological behavior (Westphal and Lamszus, 2011). At the molecular level, glioma development is tightly linked to a range of genetic and epigenetic factors, including gene mutations, chromosomal abnormalities, and dysregulation of gene expression (Zhou et al., 2018; Zhao et al., 2021). Despite significant advancements in glioma research in recent years, precise molecular mechanisms and effective therapeutic strategies remain to be fully elucidated (Pan et al., 2021; Cun et al., 2023). Effective drugs for treating GBM remain limited, with temozolomide—discovered around 20 years ago—being one of the few options available (Hegi et al., 2005), however, the patient’s survival time remains around 15 months. Given the increasingly recognized role of EVEs in tumor progression, exploring their function and mechanisms in glioma could lead to the development of new therapeutic strategies for GBM treatment.

Recent studies have revealed the potential roles of EVEs in various tumors, such as their involvement in tumor microenvironment regulation and immune evasion (Vitiello et al., 2021). HERV-K knockdown induces changes in the transcription factor p53 and myc-related pathways in breast cancer cells (Zhou et al., 2016). HERV-K knockdown also suppresses pancreatic cancer cell proliferation and tumor growth by upregulating p53 and decreasing the expression of RAS and p-AKT (Li et al., 2017). However, research specifically focused on gliomas remains relatively limited. Particularly, the mechanisms by which EVEs regulate key transcription factors influencing glioma development and progression are still in their early stages of exploration. A deeper understanding of the biological functions of these EVEs and their roles in gliomas could be pivotal in developing new diagnostic and therapeutic strategies. Additionally, clarifying the interactions between EVEs and glioma development may uncover novel molecular targets, providing new directions for the treatment of this challenging malignancy.

## Methods and materials

### Cell culture

Glioma cells were maintained in DMEM (Gibco, USA) with 10% fetal bovine serum (FBS) (Gibco, USA) and 1% penicillin-streptomycin (Gibco, USA). To enrich glioma stem cells (GSCs), cells were cultured as described previously (Li et al., 2019). Cells were plated in 6 cm culture dishes with DMEM/F12 containing 1× B27 supplement (Gibco, USA), 50 ng/mL epidermal growth factor (EGF), and basic fibroblast growth factor (bFGF) (Peprotech, USA). GSCs spheres were passaged and grown in GSCs medium for enrichment. Cells were used for experiments after six passages.

### Quantitative PCR

Total RNA was extracted from glioma cells using Trizol, in accordance with the manufacturer’s guidelines. The RNA was then converted to cDNA using random primers and a cDNA synthesis kit (Thermo Scientific, USA). Quantitative PCR (qPCR) was conducted using the SYBR Green method (Thermo Scientific, USA). The relative gene expression was determined by the 2^−ΔΔCT^ method, using 18S rRNA as the reference gene for normalization.

### Flow cytometry

U251-GSC cells were transfected with siRNA validated in the literature for effectively knocking down HERV-H expression (Kudo-Saito et al., 2014). After 72 hours, the cells were fixed with 4% paraformaldehyde (PFA) for ten minutes, permeabilized with 0.3% Triton X-100 for five minutes, and then blocked with 5% FBS for one hour. Subsequently, the cells were intracellularly stained with anti-Oct4 (BioLegend, USA). After washing with flow cytometry staining buffer, and the BD LSRFortessa cell analyzer (BD Biosciences) was used to conduct flow cytometry analysis.

### Analysis of the impact of endogenous virus-related genes on tumors

CancerHERVdb is a specialized database for collecting and analyzing data related to human endogenous retroviruses (HERVs) associated with cancer (Stricker et al., 2023). To utilize this database, we accessed the search interface and entered “Brain tumor” as the cancer type to retrieve data on HERV expression related to glioblastoma. The database provides detailed information on the localization of HERVs, including their positions within the human genome and their associated cancer types. We employed the built-in visualization tools to examine the differences in HERV expression across various samples, allowing for a comprehensive analysis of HERV involvement in GBM.

### Clinical and multi-omics data analysis of glioma patients

cBioPortal offers an interactive web interface for querying, analyzing, and visualizing large-scale cancer genomics data (Brunner et al., 2022). To use cBioPortal, we selected the TCGA GBM dataset and utilized the “Query” module to perform customized queries on key genes and TCGA samples relevant to GBM. Additionally, we employed the “OncoPrint” feature to visually represent the mutations in key genes across multiple GBM samples, providing a clear and detailed overview of gene variations within the dataset. The Cancer Genome Atlas (TCGA) is a comprehensive resource containing clinical and molecular data for various cancer types (Tang et al., 2019). We accessed these data through the Genomic Data Commons (GDC) Data Portal. After registering and logging into the GDC portal, we used the web-based interface to search and filter the data. We specifically selected the GBM dataset and focused on data types such as gene expression, mutation, and copy number variation. This enabled us to conduct in-depth analyses of the genomic alterations associated with GBM. The Chinese Glioma Genome Atlas (CGGA) is a database focused on glioma genomic data from the Chinese population (Zhao et al., 2021). We accessed the data via the CGGA website, which offers user-friendly search tools. Queries were conducted based on case IDs, gene names, or pathological characteristics. Furthermore, CGGA provides visualization tools, which we used to generate graphs displaying gene expression levels, mutation frequencies, or survival analyses. This allowed for a detailed examination of the genetic and clinical features of gliomas in the Chinese population.

### Statistical analysis

Patients with RNA-seq for OCT4 expression are from TCGA and Chinese Glioma Genome Atlas (CGCA). For data that followed a normal distribution, an unpaired two-tailed Student’s *t*-test was employed to compare two groups. Differences among three or more groups were assessed using one- or two-way Anova. Gene expression correlations were analyzed using Pearson’s correlation analysis. The survival rate was estimated by the Kaplan−Meier method and log-rank test. Statistical analyses were carried out using GraphPad Prism software. The results are expressed as mean ± SD. *P <*0.05 is considered statistically significant.

## Results

### EVEs activate transcription factor OCT4 in glioblastoma

**Figure 1:**
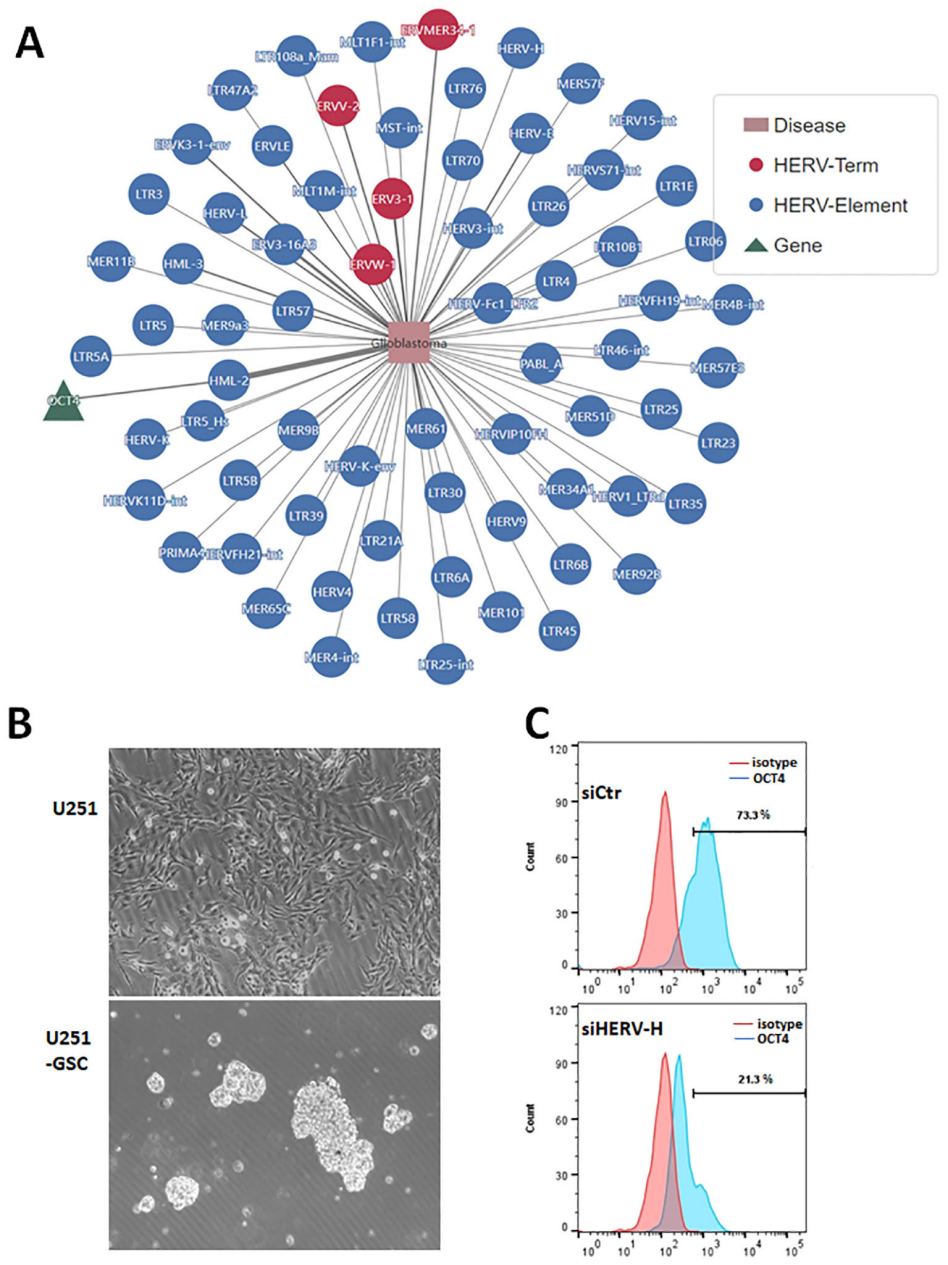
Endogenous viral elements activate transcription factor OCT4 in glioblastoma. **(A)**. Analysis of genes associated with glioblastoma using the HervD Atlas database. The pink rectangles represent diseases, the green triangles represent genes, and the red and blue circles represent HERV Terms and Elements, respectively. The lines indicate potential regulatory relationships between them. **(B)**. Glioblastoma stem cells (U251-GSC) isolated from the U251 cell line growing as neurospheres. The image below shows glioma stem cells growing into neurospheres. **(C)**. U251-GSC cells were transfected with control siRNA (siCtr) and HERV-H siRNA (siHERV-H) for 72 hours. OCT4 expression was analyzed using immunofluorescence staining with an OCT4 antibody, followed by flow cytometry detection of OCT4 levels. An isotype control was used as the corresponding control for the OCT4 antibody.

We analyzed the potential roles of endogenous viral elements in different cancers using data from the CancerHERVdb database (Stricker et al., 2023). Our findings revealed that brain tumors are significantly regulated by endogenous viral elements (**Supplementary Figure 1**). Subsequently, we utilized the HervD Atlas database to investigate the activation of key genes in glioblastoma by endogenous viral elements (Paces et al., 2004; Li et al., 2024). Our analysis identified that OCT4, also known as POU5F1, is a key gene in glioblastoma activated by endogenous viral elements (**Figure 1A**). OCT4 is a well-known transcription factor involved in maintaining stem cell pluripotency and has been potentially implicated in glioma (Nayak and Singh, 2021). We isolated and cultured glioblastoma stem cells from the U251 glioblastoma cell line, obtaining U251-GSCs (glioblastoma stem cells) (**Figure 1B**). According to the research, HERV-H is the most prevalent and enriched in microvesicles within these GBM cells, followed by HERV-C, and HERV-K6 (Balaj et al., 2011). To investigate the effect of HERV-H on OCT4 expression, we transfected U251-GSC cells with either control siRNA (siCtr) or siRNA specifically targeting HERV-H (siHERV-H). After 72 hours, OCT4 expression was assessed through flow cytometry, and the results indicated that silencing HERV-H significantly reduced OCT4 expression in U251-GSC cells (**Figure 1C**). Therefore, we hypothesize that the activation of OCT4 by EVEs contributes to the progression of GBM. However, the impact of EVEs-induced OCT4 on GBM is not yet fully understood.

### Association between the OCT4 expression and mutations of key genes that regulate glioma

**Figure 2:**
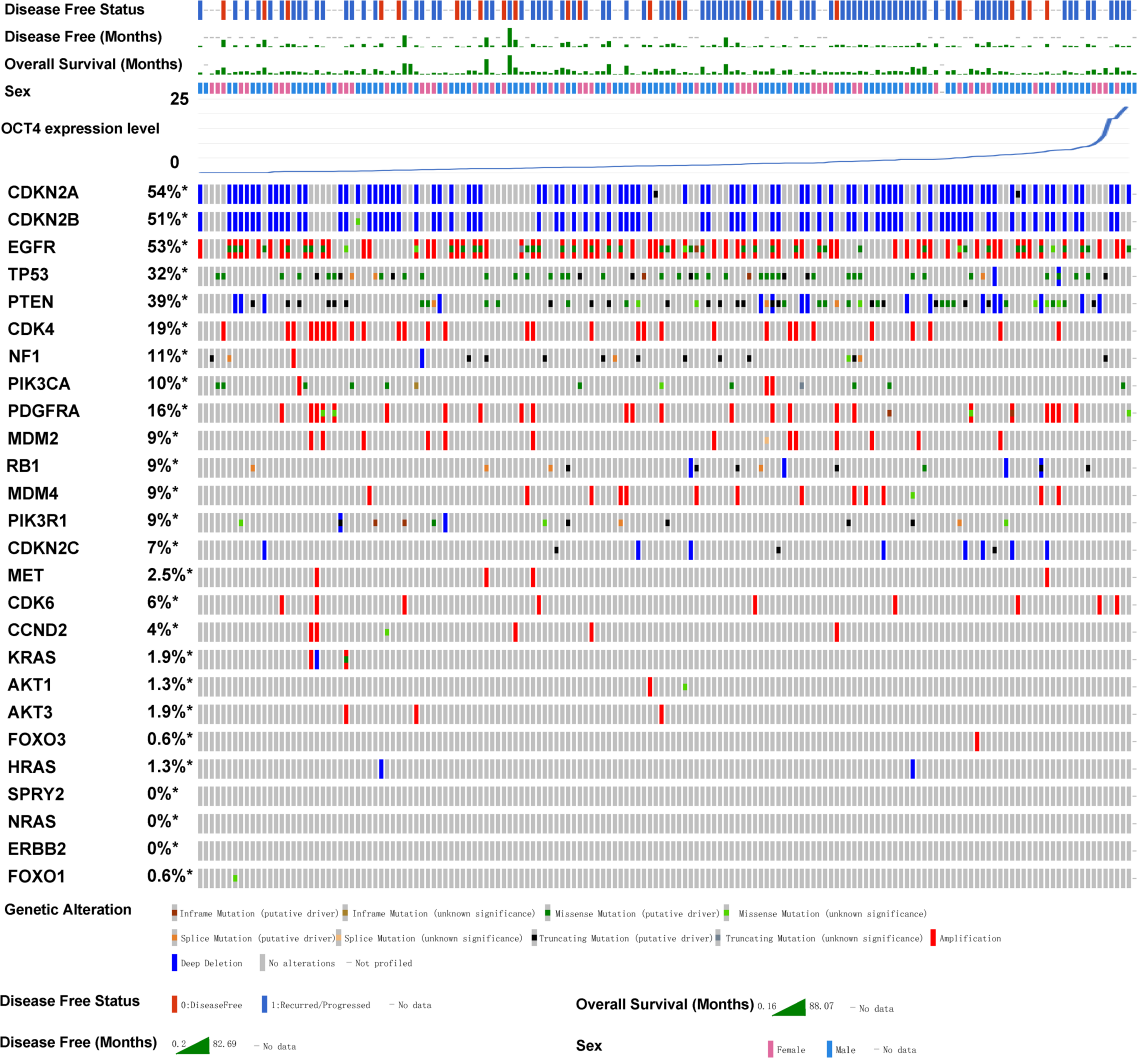
OCT4 expression is associated with key glioblastoma gene mutations in glioma patients. The relationship between OCT4 expression and mutations in key glioblastoma genes was analyzed using cBioPortal. Genomic alterations such as copy number alterations (deep deletions and amplification), mutations such as missense, splice and truncating mutations, and up and down-regulation of mRNA are summarised by glyphs and coding. The cases are represented according to alterations. The analysis provides a summary of genomic alterations (legend) (rows) affecting individual patients (columns). The mutational frequency is labelled on the left in percentage.

Subsequently, we utilized the TCGA database to analyze GBM samples from 273 patients in cBioPortal (Cerami et al., 2012; Zhou et al., 2018). We aim to analyze whether OCT4 is related to the clinical phenotypes of gliomas. Therefore, we conducted a detailed analysis of the relationship between various clinically associated glioma genes and OCT4 expression levels. The GBM samples were sorted based on the expression levels of OCT4. Our results revealed that OCT4 expression was associated with mutations in key genes that regulate glioma (**Figure 2**). For example, the well-known cancer-related gene CDK4 shows a higher frequency of copy number variations when OCT4 expression is low, while CDK4 expression decreases when the patient’s OCT4 gene expression is upregulated. Similarly, the mutation trends of the NF1 and PIK3CA genes correlate with OCT4 expression in a manner similar to CDK4 copy number variations. Meanwhile, the mutation frequency of MDM4 and RB1 is inversely related to the levels of OCT4 expression in patients. Although other genes showed only a modest correlation, future studies with larger patient samples may reveal clearer trends. Nevertheless, these observations still indicate that many key tumor-related genes are closely associated with OCT4 expression, which is a significant clinical characteristic. This may also suggest that OCT4 expression may be linked to alterations in key oncogenic pathways.

### OCT4 expression is significantly associated with patient prognosis and glioma malignancy grading in patients with glioma

**Figure 3:**
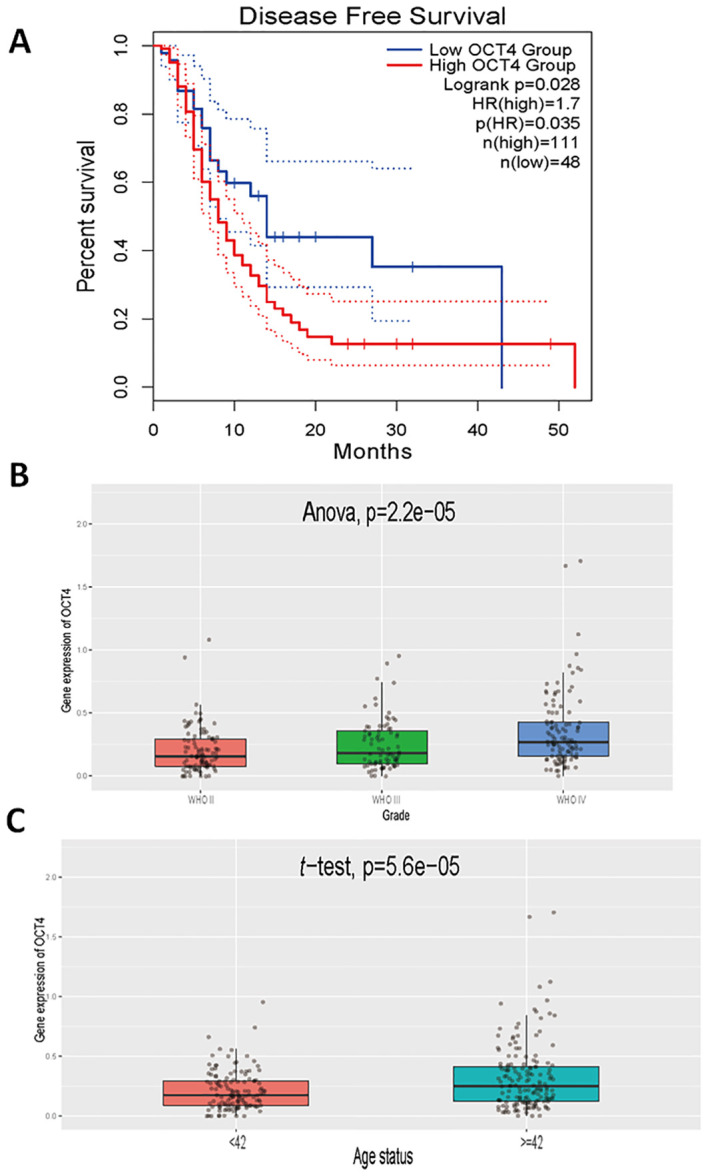
Clinical relevance of OCT4 expression in glioblastoma patients. **(A)**. We performed a Kaplan-Meier survival analysis to compare overall survival rates between glioblastoma patients exhibiting high and low expression levels of OCT4. This analysis utilized survival data sourced from The Cancer Genome Atlas (TCGA), which includes comprehensive clinical and genomic information on glioblastoma patients. The survival curves were constructed to visualize and assess the impact of OCT4 expression on patient prognosis. **(B)**. We performed a differential expression analysis to evaluate variations in OCT4 expression levels among glioblastoma patients classified into different World Health Organization (WHO) grades: Grade II, Grade III, and Grade IV. This analysis was based on data from the China Glioma Genome Atlas (CGCA), which provides a rich dataset of glioblastoma patient samples categorized by tumor grade. The goal was to determine if OCT4 expression correlates with the severity of the disease. **(C)**. We performed a differential expression analysis to investigate OCT4 expression levels among glioblastoma patients stratified by age categories. The analysis was carried out using data from the China Glioma Genome Atlas (CGCA). This study aimed to identify any significant variations in OCT4 expression that might be associated with different age groups, potentially revealing age-related patterns in OCT4 expression and its implications for glioblastoma progression and patient outcomes.

To further confirm the role of EVEs-activated OCT4 in GBM, we also analyzed prognosis and other clinically relevant outcomes. The results revealed that patients with high OCT4 expression had significantly worse prognosis compared to patients with low OCT4 expression in the TCGA GBM dataset (**Figure 3A**). Additionally, we observed that higher grades of GBM were associated with increased OCT4 expression, indicating a correlation between OCT4 expression and malignant progression of glioblastoma (**Figure 3B**). Finally, we found that older patients exhibited higher OCT4 expression levels, suggesting that advanced age is associated with elevated OCT4 expression (**Figure 3C**). This suggests that aging may be associated with increased OCT4 activity, potentially influencing the progression of GBM in older individuals. These results demonstrate that high OCT4 expression in glioblastoma patients correlates with significantly worse prognosis, higher tumor grades, and advanced age.

### SOX2 and OCT4 are co-expressed at high levels in glioblastoma stem cells

**Figure 4:**
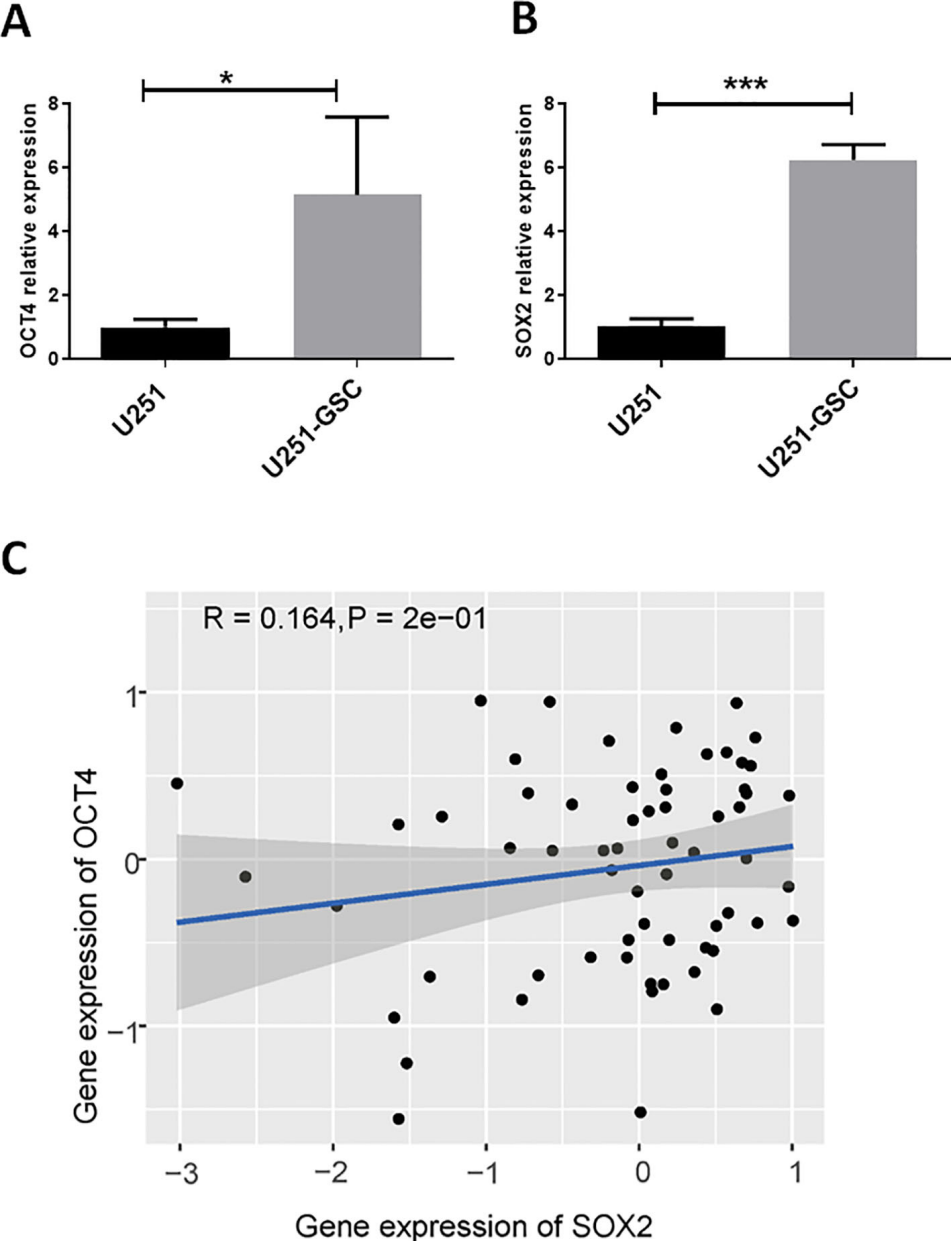
OCT4 and SOX2 are up-regulated in glioblastoma stem cells. **(A)**. We performed a quantitative polymerase chain reaction (qPCR) analysis to quantify the expression levels of OCT4 in both U251 glioblastoma cells and their derived glioblastoma stem cells (U251-GSC). The qPCR data indicated a statistically significant difference in OCT4 expression between these two cell types, with a p-value of less than 0.05. *P<0.05. **(B)**. We performed a quantitative polymerase chain reaction (qPCR) to measure the expression levels of SOX2 in U251 glioblastoma cells and U251-GSC (glioblastoma stem cells). The analysis revealed a highly significant difference in SOX2 expression between the two cell types, with a p-value of less than 0.001. ***P<0.001. C. The Pearson correlation analysis of SOX2 and OCT4 gene expression in glioma patients from the CGGA database is shown. The P-value and R-value are displayed in the figure to indicate the strength and significance of the correlation.

OCT4, as a stemness-related regulatory gene, may play a crucial role in glioblastoma stem cells. Compared to the U251 cell line, OCT4 expression was significantly upregulated in U251-GSCs (**Figure 4A**). Additionally, the expression of another key gene, SOX2, which is important for glioblastoma stem cells, was also significantly upregulated (**Figure 4B**). Analysis of data from the CGCA database reveals a significant positive correlation between OCT4 and SOX2 expression in GBM patients (**Figure 4C**). These findings suggest that OCT4, activated by endogenous viral elements, may play a crucial role in the malignant progression of glioblastoma. We also hypothesize that endogenous viral elements might regulate other transcription factors associated with glioma stem cells, which could be an avenue for future research.

## Discussion

Endogenous viral elements (EVEs) have emerged as significant players in cancer biology (Jansz and Faulkner, 2021). EVEs can serve as binding sites for transcription factors, thereby directly or indirectly modulating the expression of nearby genes. This ability to influence gene activity underscores the complexity of EVEs-mediated gene regulation. For instance, EVEs have been shown to contribute thousands of binding sites for critical pluripotency factors such as OCT4 and Nanog in embryonic stem cells (Kunarso et al., 2010). This interaction highlights how EVEs can integrate into regulatory networks that govern cellular processes and maintain stem cell characteristics. Our results indicate that the downregulation of HERV-H leads to a decrease in OCT4 expression in glioma stem cells. HERVs contain long terminal repeats (LTRs) that can serve as binding sites for various transcription factors. Studies have shown that the HERV-H LTR functions as an enhancer, and knocking down HERV-H in human embryonic stem cells (hESCs) results in the downregulation of pluripotency markers, including OCT4, SOX2, and NANOG (Lu et al., 2014). Following OCT4 knockdown, the activity of the HERV-H LTR enhancer is reduced, suggesting a feedback loop in which OCT4, as a transcription factor, binds to the regulatory regions of certain HERVs, mutually enhancing the expression of both OCT4 and HERVs (Lu et al., 2014). Moreover, HERVs may induce changes in DNA methylation patterns and histone modifications (Hurst and Magiorkinis, 2017), creating a chromatin environment conducive to OCT4 expression. This epigenetic reprogramming sustains cancer stem cell-like properties in glioma, promoting tumor aggressiveness and resistance to treatment. Recent study also suggests that HERV-K overexpression in glioblastoma contributes to maintaining the cancer stem cell phenotype (Shah et al., 2023). The regulatory mechanisms between EVEs and OCT4 warrant further investigation.

To date, there has been a lack of studies on the role of EVEs in regulating transcription factors and influencing glioblastoma progression. Our study highlights the significant role of endogenous viral elements (EVEs) in glioblastoma progression through the activation of OCT4, a key transcription factor involved in stemness and malignancy. Moreover, OCT4 expression was associated with mutations in key genes that regulate glioma indicates that OCT4 may influence critical oncogenic pathways. Our study highlights the clinical significance of OCT4 by demonstrating its association with poorer prognosis, older age, and higher tumor grades in glioma patients. Additionally, our results revealed that high OCT4 expression in glioma stem cells is accompanied by increased expression of another essential glioma stem cell gene, SOX2 (Guo et al., 2021). Positive correlation between OCT4 and SOX2 expression in GBM patients suggests an important role of OCT4 and SOX2 in the pathology of glioma.

In this study, our findings suggest that the activation of OCT4 by Endogenous Viral Elements (EVEs), particularly Human Endogenous Retroviruses (HERVs), may play a critical role in GBM progression. We identified a significant association between OCT4 expression levels and glioblastoma multiforme (GBM) prognosis, tumor grading, and patient age. The study underscores the importance of OCT4 as not only a marker of poor prognosis but also a potential therapeutic target. Targeting this axis could disrupt the feedback loop that maintains cancer stem cells in GBM. Understanding the relationship between OCT4 and EVEs activation opens up potential therapeutic avenues. Potential strategies might include using small molecule inhibitors to block the binding of OCT4 to HERVs or using epigenetic drugs to silence EVE activity, thereby reducing OCT4 expression and weakening the CSC population in the tumor (Hosseiniporgham and Sechi, 2023; Dai et al., 2024). It is also necessary to further investigate whether antiretroviral inhibitors can suppress the expression of EVEs, as these antiretroviral drugs could be ideal candidates for the treatment of GBM (Tyagi et al., 2017). Future studies should focus on elucidating the precise molecular mechanisms through which EVEs, especially HERVs, activate OCT4. This could involve investigating the role of specific transcription factors, chromatin modifications, and other epigenetic factors that mediate this activation (Gao et al., 2021). Our study primarily focuses on cellular-level experiments and expression analysis in clinical patients. To translate these findings into clinical applications, *in vivo* studies and clinical trials are needed to assess EVE-OCT4 pathway in GBM. These studies could help determine the potential benefits of combining such therapies with existing treatment modalities.

## Data Availability

Publicly available datasets were analyzed in this study. These data can be found here: HervD Atlas: https://ngdc.cncb.ac.cn/hervd/; CancerHERVdb: https://erikstricker.shinyapps.io/cancerHERVdb/; TCGA: https://www.cancer.gov/ccg/research/genome-sequencing/tcga; CGGA: http://www.cgga.org.cn.

## Supplementary material

Supplementary Figure 1 HERV expression in various tumor types. Expression levels of Human Endogenous Retroviruses (HERVs) across various tumor types, based on data from the CancerHERVdb database. The y-axis represents the HERV expression, while the x-axis lists different types of cancers, ordered from highest to lowest HERV expression.
