# Supplementary figures and images for: Impact of endogenous viral elements on glioma clinical phenotypes by inducing OCT4 in the host
Source: Front Cell Infect Microbiol. 2024 Nov 11;14:1474492. doi: 10.3389/fcimb.2024.1474492 (PMC11586349; doi:10.3389/fcimb.2024.1474492)

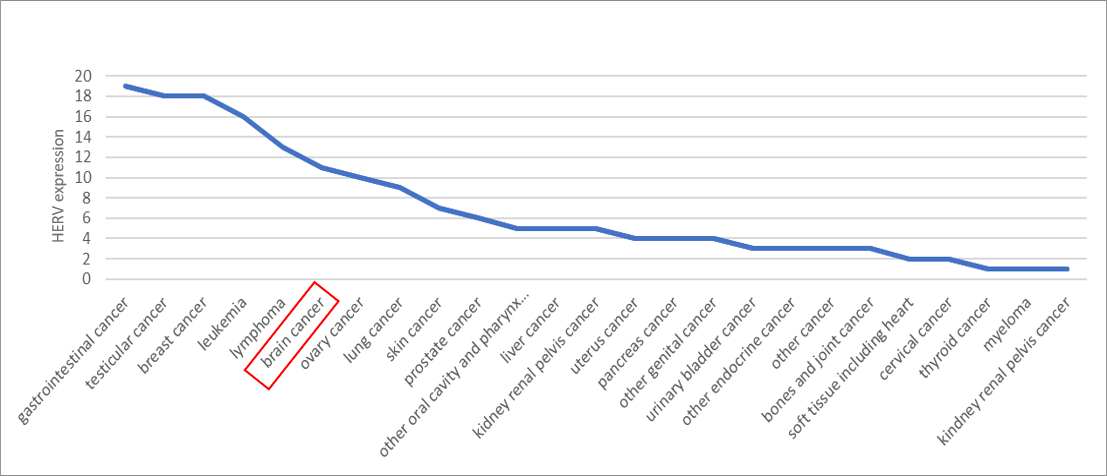

Supplement: Supplementary Figure 1 — HERV expression in various tumor types. Expression levels of Human Endogenous Retroviruses (HERVs) across various tumor types, based on data from the CancerHERVdb database. The y-axis represents the HERV expression, while the x-axis lists different types of cancers, ordered from highest to lowest HERV expression. [file Image1.tif]
